# Mental illness and socio-economic situation of women and men diagnosed with gambling disorder (GD) in Sweden - nationwide case-control study

**DOI:** 10.1371/journal.pone.0274064

**Published:** 2022-10-26

**Authors:** Louise Larsson, Anders Håkansson

**Affiliations:** 1 Örebro University, School of Medical Sciences, Örebro, Sweden; 2 Region Skåne, Malmö Addiction Center, Competence Center Addiction, Malmö, Sweden; 3 Faculty of Medicine, Department of Clinical Sciences Lund, Psychiatry, Lund University, Lund, Sweden; Yale University, UNITED STATES

## Abstract

The present study aimed to compare men and women with gambling disorder (GD) regarding presence of psychiatric comorbidity and socio-economic vulnerability, and to examine whether these factors appear before or after the gambling disorder. This is a retrospective case-control study, based on registers from The National Board of Health and Welfare and Statistics Sweden. A total of 3592 adults with GD were matched with two controls based on age and gender, including a total of 10776 individuals in the study. The study included psychiatric comorbidity through the presence of relevant diagnostic codes or pharmacological codes, and socio-economic vulnerability data through the presence of unemployment, social welfare payments and sickness/activity/rehabilitation compensation. Time between GD and psychiatric comorbidity/socio-economic vulnerability was calculated by subtracting dates between diagnoses/first incidence of socio-economic vulnerability factor and GD diagnosis. Women with GD were more likely to have a psychiatric comorbidity, compared to men. Overall, women were also more likely to receive their psychiatric diagnosis prior to GD diagnosis, while men were more likely to receive the diagnoses concurrently. Social welfare payments, and sickness support were more common among women, while there was no difference in unemployment between genders. Women were also more likely to receive sickness/activity/rehabilitation compensation prior to GD, than men who were more likely to receive these types of support after GD diagnosis. In conclusion, women appear to be at higher risk of psychiatric comorbidity and socio-economic vulnerability alongside GD. They are in general also more likely to receive have their psychiatric and psycho-social problems identified prior to GD, than men who are more likely to receive diagnoses concurrently.

## Introduction

Gambling disorder (GD) is a psychiatric condition which affects approximately 0.6% of the adult Swedish population, and another 3.6% are at risk [1]. GD is considered an addictive disorder according to the DSM-5, which thereby constituted a somewhat new approach, as it previously has been regarded as an impulse control disorder [2]. Research shows that the reward system in the brain, for instance, is activated in ways resembling that of substance addiction [3].

Gambling is a popular activity for both men and women. In age groups 16–84 years, one study conducted on a Swedish population found that approximately 32% men and 21% of women had gambled in the past month [4]. The most active age groups were 45–64 and 65–84 years, where gambling prevalence reached 34%. However, these groups were less likely to be problem gamblers, which was the most common in age groups 25–44, reaching 1.9% of the population [1].

While new research shows that women are still in minority, the women who do gamble may be just as much at risk for GD as men [5]. Despite the fact that GD is overrepresented in men, recent studies indicate that women are at higher risk of psychological distress and over-indebtedness, indicating that they may be at higher risk of severe gambling-related consequences [6].

Psychiatric comorbidity such as depression and anxiety disorders also seem to precede the gambling disorder for women, but not as much for men, indicating that women use gambling as a strategy to cope with other issues including psycho-social problems [7]. The type of psychiatric comorbidity also differs between genders; one study showed that women are more likely to have co-morbid affective or anxiety disorders, while there is no difference in substance use disorders between genders [8].

Financial issues connected to gambling highlight that lower socio-economic status is a risk factor [9]. People with higher incomes seem to spend more money on gambling, while those with lower incomes proportionally spend a higher percentage of their income [10]. Many studies use income, education level and working status as markers for socio-economic situation, but few studies have investigated other socio-economic factors, such as sickness compensation or social welfare payments.

For both socio-economic vulnerability and psychiatric comorbidities, it is poorly understood whether these factors appear before, concurrently with, or after GD, as few studies have been able to map longitudinal patterns, especially regarding socio-economic situation. This is an important aspect as it contributes to a better understanding of GD, and could potentially help clinicians provide better preventative actions for both women and men with GD.

The aim of this study was to investigate gender differences in gambling disorder regarding psychiatric comorbidity as well as socio-economic vulnerability, and to examine whether psychiatric comorbidity or socio-economic vulnerability appears before, concurrently with, or after a diagnosis of gambling disorder.

## Material and methods

**Fig 1:**
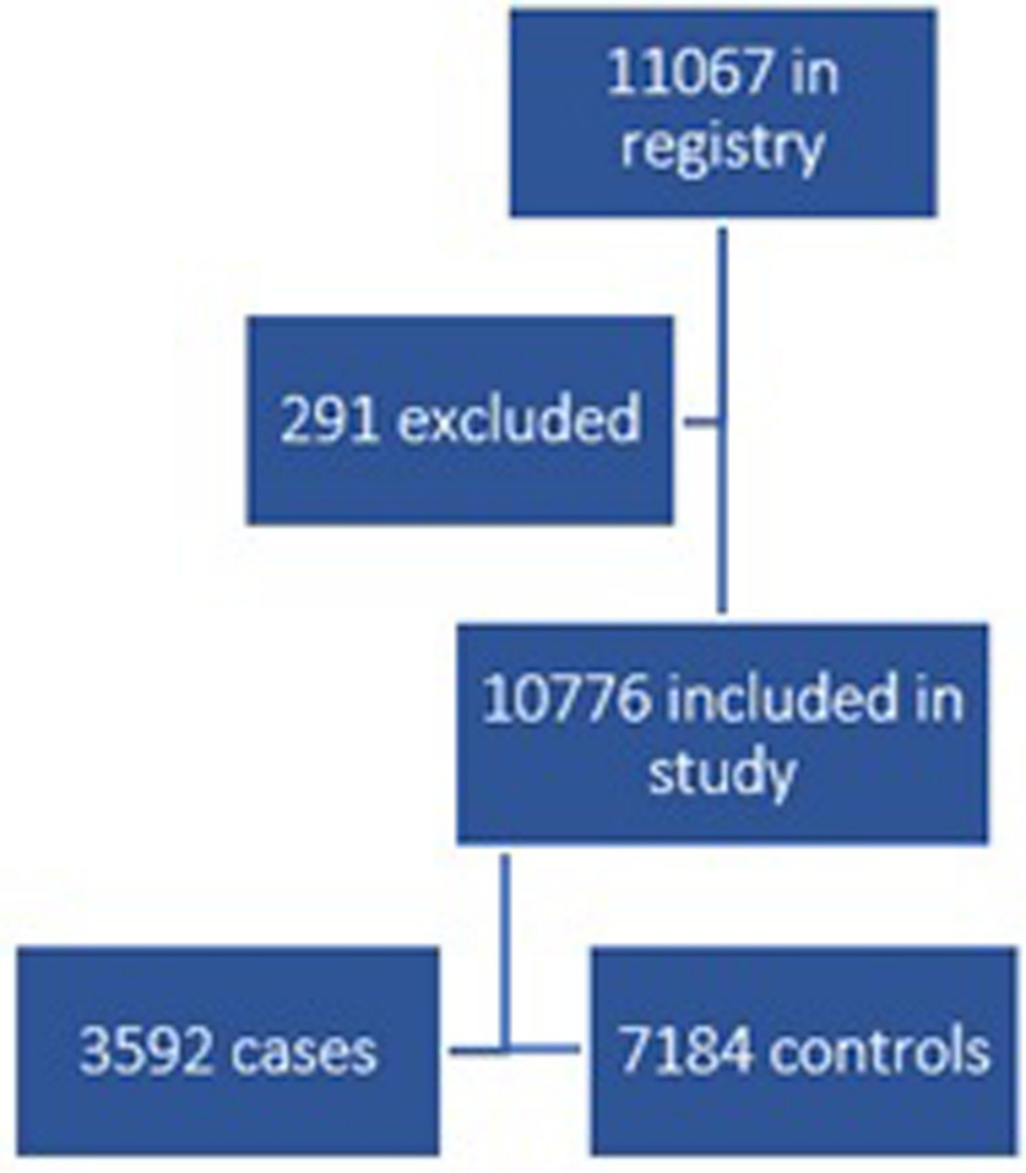
Flow chart over inclusion criteria for the study. 291 individuals were excluded due to being under 18 years of age. In total, 10776 individuals were included.

The study was a retrospective case-control study, where information was retrieved from three registers: the National Patient Register and the Swedish Prescribed Drug Register, retrieved from the National Board of Health and Welfare, and the Longitudinal integrated database for health insurance and labour market studies (LISA) register, retrieved from Statistics Sweden. The data retrieved included all individuals with a diagnosis of GD in Swedish specialized healthcare (ICD-10 code F63.0, referred to as ‘pathological gambling’) at any time between 2005–2019 for all registers except for the LISA register, which covers the years 2005–2018, and for each individuals with a GD, two control subjects were randomly drawn from the general population. Control subjects were to have no diagnosis of GD in their lifetime (but may have had any other diagnosis). The two control subjects selected for every GD patient were gender- and age-matched (same gender and the same age, in years). These controls were randomly selected from the Statistics Sweden population register. In total, 11067 individuals were included in the material, of which 3869 have a GD diagnosis. Individuals below the age of 18 were excluded (Fig 1).

Psychiatric comorbidity was defined by the presence of a mental health diagnosis, based on in- and outpatient visits in the National Patient Register, and medical prescriptions for psychiatric medications, respectively. The latter was also included because data from primary care visits could only be retrieved from the Swedish Prescribed Drug Register, as primary care diagnoses are not included in the Patient Register. The specific sub-groups of psychiatric comorbidities examined in this study comprised alcohol use disorder (ICD-10 codes F10) and other substance use disorders (F11-F19), psychotic disorders (F20-F29), affective disorders (F30-F39), anxiety disorders, phobias, PTSD, obsessive-compulsive disorders and dissociative syndromes (F40-F49), neuropsychiatric disorders/behavioural and emotional disorders with onset usually occurring in childhood and adolescence (F90-F98), and mental disorder not otherwise specified (F99). Medical prescriptions analysed were drugs for alcohol dependence (ATC code N07BB), antidepressants (N06A), sedatives (N05B) and sedatives and hypnotics (N05C). Socio-economic status was defined by any incidence of unemployment, social welfare payments, or sickness/activity/rehabilitation compensation in the LISA register. There was no minimum limit for how long an individual had any of these factors. However, sickness compensation requires that the individual is permanently unable to work, activity compensation requires an estimated sickness duration of at least one year, and rehabilitation compensation at least 60 days.

The psychiatric comorbidities and socio-economic variables in the GD group were compared to controls, as well as compared between genders. Furthermore, for psychiatric comorbidities and socio-economic factors, an analysis of the GD group assessed whether they received the GD diagnosis before, concurrently with, or after the psychiatric comorbidity diagnosis/socio-economic vulnerability factor. If a patient had received another psychiatric diagnosis within 180 days of their GD diagnosis, they were considered to have received the diagnoses concurrently. For socio-economic factors, this interval was extended to 365 days as the registers only provided yearly information on those variables.

The relationships between psychiatric comorbidity as well as socio-economic status and GD in women and men were analysed through crosstabulations; first, a comparison of the prevalence of mentioned factors between the GD group and the control group for each gender, and second, a comparison between men and women in the GD group. The GD and control groups were compared unmatched to each other. From these analyses, odds ratios and 95% confidence intervals were retrieved. To investigate the time between GD and psychiatric comorbidity/socio-economic factors, Chi-square test for homogeneity was first performed to determine if there were any gender differences. A subsequent post-hoc test using Bonferroni correction for p value of 0.05/3 = 0.0167 was then performed when comparing women and men in the GD group, investigating whether they received their psychiatric comorbidity/socio-economic factor diagnosis prior to, concurrently with, or after their GD diagnosis. All statistical analyses were performed with IBM SPSS Statistics version 28.0.

The study has been ethically approved by the Swedish Ethical Review Authority, with registration number 2019–01559. This study will only be using information from the registers, and the data has already been retrieved by the research group located at Malmö Addiction Center. All names and personal data have been anonymised and coded, and the codes are not available to the research group as they are kept by the administrative authorities that provided the registers.

## Results

Mean age and distribution of women and men as well as numbers and proportions of individuals in each group with psychiatric comorbidity, drug prescription and socio-economic variables are presented in Table 1.

**Table 1:** Baseline characteristics and proportions of psychiatric comorbidity and socio-economic factors in register data from patients with gambling disorder (GD) and in controls (age- and gender-matched individuals randomly included from the general population without GD).

	GD group (N = 3592)			Controls (N = 7184)		
	Men	Women	p value	Men	Women	p value
**N (%)**	2791 (78)	801 (22)		5582	1602	
**Age (CI 95%)**	34 (34–35)	40 (39–41)	***			
**Alcohol use disorder, total (%)**	792 (28)	208 (21)	0.180	223 (4)	42 (3)	*
GD before (%)	384 (49)	120 (58)	0.018			
Concurrentlyi(%)	207 (26)	34 (16)	**			
GD after (%)	201 (25)	54 (26)	0.864			
**Other substance use disorders, total (%)**	678 (24)	215 (27)	0.141	181 (3)	39 (2)	0.055
GD before (%)	215 (32)	69 (32)	0.917			
Concurrentlyi(%)	218 (32)	59 (27)	0.193			
GD after (%)	245 (36)	87 (41)	0.252			
**Psychotic disorders, total (%)**	180 (6)	37 (5)	0.055	29 (0.5)	3 (0.2)	0.078
GD before (%)	50 (28)	11 (30)	0.810			
Concurrentlyi(%)	75 (42)	14 (38)	0.666			
GD after (%)	55 (31)	12 (32)	0.822			
**Affective disorders, total (%)**	1327 (48)	534 (67)	***	313 (6)	140 (9)	***
GD before (%)	276 (21)	99 (19)	0.272			
Concurrentlyi(%)	521 (39)	173 (32)	**			
GD after (%)	530 (40)	262 (49)	***			
**Anxiety disorders, PTSD, phobias, total (%)**	1517 (54)	560 (70)	***	458 (8)	208 (13)	***
GD before (%)	358 (24)	133 (24)	0.943			
Concurrentlyi(%)	602 (40)	197 (35)	0.061			
GD after (%)	557 (37)	230 (41)	0.069			
**Neuropsychiatric disorders, total (%)**	517 (19)	171 (21)	0.232	170 (3)	33 (2)	*
GD before (%)	186 (36)	66 (39)	0.538			
Concurrentlyi(%)	141 (27)	30 (18)	*			
GD after (%)	190 (37)	75 (44)	0.098			
**Mental disorder, not otherwise specified, total (%)**	120 (4)	40 (5)	0.375	23 (0.4)	8 (0.5)	0.640
GD before (%)	46 (38)	8 (20)	0.034			
Concurrentlyi(%)	33 (28)	9 (23)	0.534			
GD after (%)	41 (34)	23 (58)	**			
**Total psychiatric comorbidity (%)**	2224 (80)	701 (88)	***	845 (15)	296 (18)	**
GD before (%)	481 (17)	144 (18)	0.628			
Concurrentlyi(%)	1349 (48)	304 (38)	***			
GD after (%)	960 (34)	353 (44)	***			
**Use of antidepressants, total (%)**	363 (13)	188 (23)	***	657 (12)	249 (15)	***
GD before (%)	32 (5)	11 (4)	0.775			
Concurrentlyi(%)	149 (23)	21 (8)	***			
GD after (%)	476 (73)	217 (87)	***			
**Use of sedatives (N05B), total (%)**	1416 (51)	606 (76)	***	917 (16)	453 (28)	***
GD before (%)	262 (19)	68 (11)	***			
Concurrently (%)	229 (16)	78 (13)	0.058			
GD after (%)	925 (65)	460 (76)	***			
**Use of sedatives and hypnotics (N05C), total (%)**	1481 (53)	600 (75)	***	873 (16)	424 (27)	***
GD before (%)	256 (17)	64 (11)	***			
Concurrentlyi(%)	317 (21)	94 (16)	**			
GD after (%)	908 (61)	442 (74)	***			
**Use of drug treatments for alcohol use disorder, total (%)**	521 (19)	168 (10)	0.144	17 (0.3)	84 (5)	0.183
GD before (%)	105 (20)	40 (24)	0.312			
Concurrentlyi(%)	203 (39)	47 (28)	**			
GD after (%)	213 (41)	81 (48)	0.095			
**Social welfare payments (%)**	1392 (50)	452 (56)	**	1031 (18)	254 (16)	*
GD before (%)	155 (11)	46 (10)	0.570			
Concurrently (%)	151 (11)	51 (11)	0.797			
GD after (%)	1086 (78)	355 (79)	0.815			
Sickness compensation**** (%)	635 (23)	348 (43)	***	347 (6)	188 (12)	***
GD before (%)	151 (24)	52 (15)	**			
Concurrently (%)	53 (8)	33 (10)	0.547			
GD after (%)	431 (68)	263 (76)	*			
**Unemployment (%)**	1073 (38)	307 (38)	0.952	1354 (24)	436 (27)	*
GD before (%)	148 (14)	35 (11)	0.276			
Concurrently (%)	67 (6)	14 (5)	0.268			
GD after (%)	858 (80)	258 (84)	0.109			

*p<0.05,

**p<0.01,

***p<0.001 for all analyses except for subcategories GD before, concurrently, and GD after, where *p <0.0167 with Bonferroni correction. CI = confidence interval. Age is expressed as a mean. Parentheses represent percentages of individuals who received a diagnosis, prescription or socio-economic variable within the time range 2005–2019 (2018 for socio-economic variables). Subcategories GD before, concurrently, and GD after represent the time aspect between GD and a psychiatric comorbidity or prescription. **** Sickness compensation also encompasses activity and rehabilitation compensation.

### Analysis of psychiatric comorbidity

**Fig 2:**
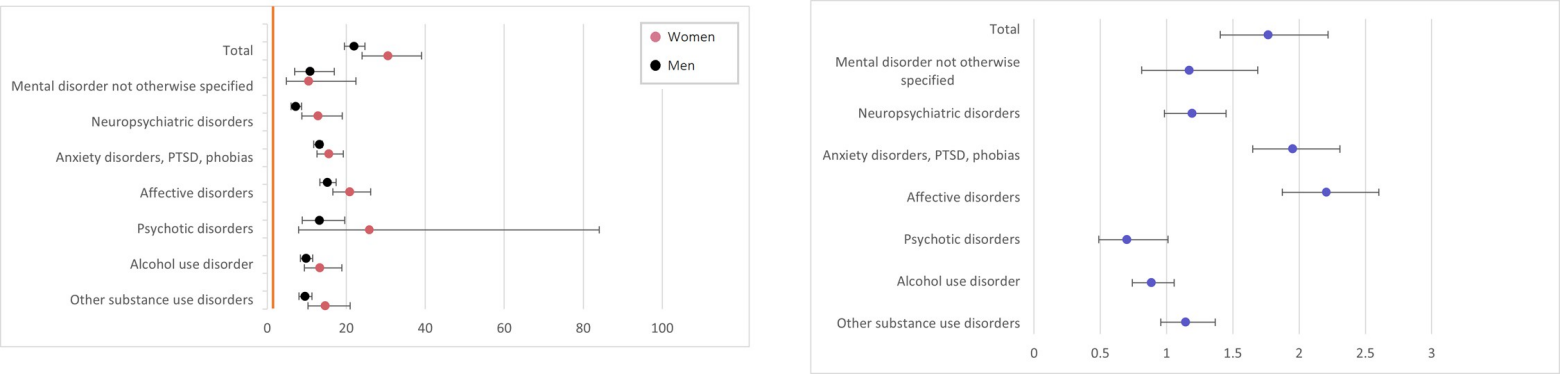
**A.** Odds ratios for women and men in the GD group having another psychiatric diagnosis, compared to controls. Orange line accentuates the odds ratio for controls (baseline). **B.** Odds ratios for women having another psychiatric diagnosis, compared with men in the GD group. Orange line accentuates the odds ratio for men (baseline).

For all tested psychiatric disorders, the GD group had at least a two-fold increase in odds compared to controls (Fig 2A). When comparing gender, women in the GD group had overall higher odds of having any psychiatric diagnosis compared to men. However, large differences were seen between diagnostic groups; for affective and anxiety disorders, women were overrepresented. An overrepresentation of women for neuropsychiatric disorders approached statistical significance, whereas for psychotic disorders and alcohol use disorder, there was a tendency towards men having higher odds, however these findings were not statistically significant (Fig 2B).

**Fig 3:**
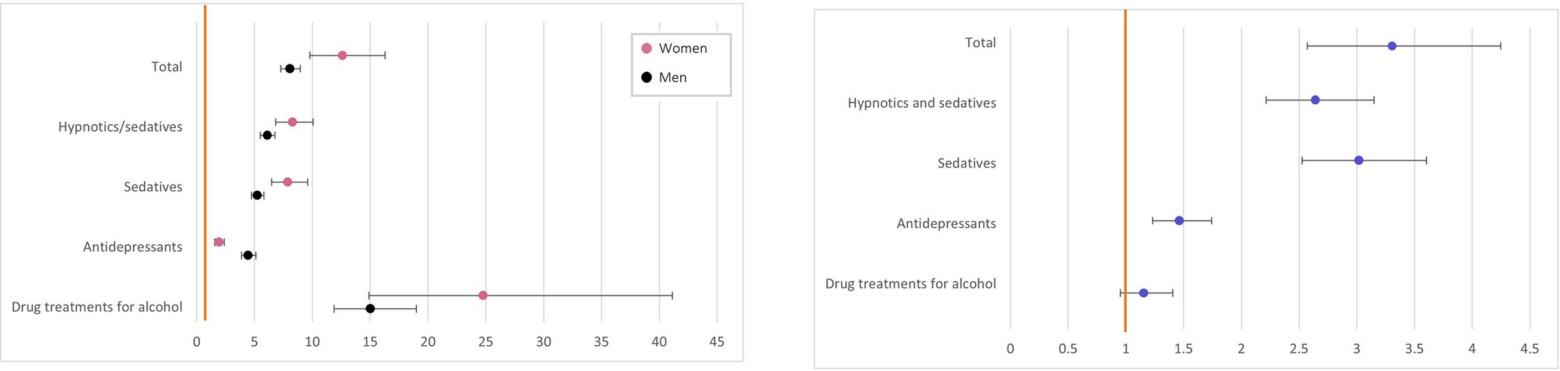
**A.** Odds ratios for women and men in the GD group being prescribed a psychiatric drug treatment, compared to controls. Orange line accentuates the odds ratio for controls (baseline). **B**. Odds ratios for women in the GD group being prescribed a psychiatric drug, compared to men. Orange line accentuates the odds ratio for men (baseline).

The GD group had higher odds of having a prescription of any of the tested psychiatric medications compared to controls, see Fig 3A. For all groups, the differences were statistically significant. When comparing gender differences, women had significantly higher odds for having a prescription of hypnotics, sedatives and/or antidepressants, see Fig 3B. Drug treatments for alcohol use disorder showed that women had a tendency towards higher odds compared to men, although this finding was not statistically significant.

### Analysis of time between diagnoses and socio-economic factors

**Fig 4:**
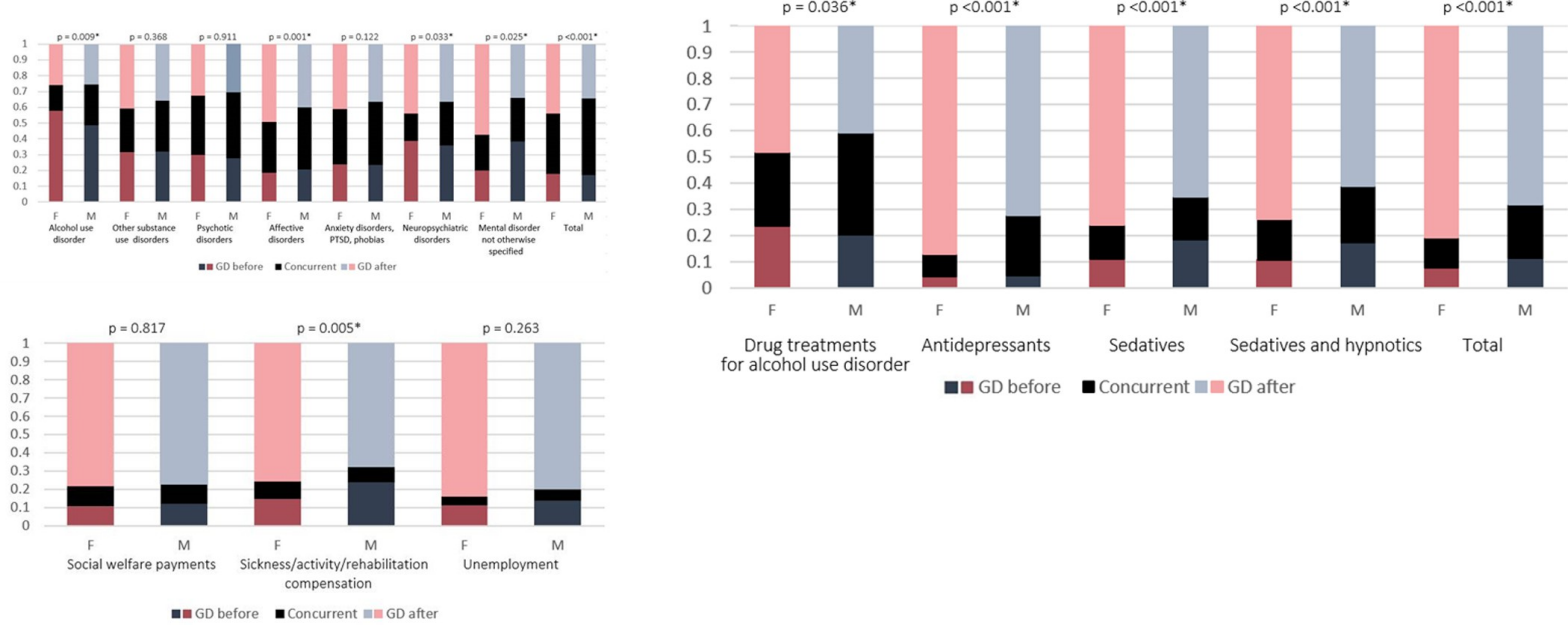
**A.** Proportions of how many of men and women with a psychiatric comorbidity had their GD diagnosis before, after or concurrently with the psychiatric comorbidity, represented as proportions of the total population who had a GD diagnosis and a specific psychiatric comorbidity. M stands for males, F females. p value represents statistical differences between women and men, asterisks mark statistically significant values. **B**. Different types of prescriptions and whether it was more common for them to be prescribed before, after, or concurrently with a GD diagnosis, represented as proportions of the total population of women or men who had a specific prescription. M stands for males, F females. p value represents statistical differences between women and men, asterisks mark statistically significant values. **C.** Proportions of how many of men and women with either social welfare payments, sickness/activity/rehabilitation compensation or unemployment had their GD diagnosis before, after or concurrently with the socio-economic factor, represented as proportions of the total population who had a GD diagnosis and a specific socio-economic factor. M stands for males, F females. p value represents statistical differences between women and men, asterisks mark statistically significant values.

Regarding whether GD appears before or after a psychiatric comorbidity, differences between diagnoses and genders are presented in Fig 4A. Four groups showed statistically significant differences with respect to gender; alcohol use disorder, affective disorders, neuropsychiatric disorders, and mental disorder not otherwise specified. In Fig 4B, a similar analysis has been performed on drug prescriptions, where there are statistically significant differences between genders for all prescriptions. For socio-economic factors, there were only statistically significant differences between genders for sickness/activity/rehabilitation compensation, see Fig 4C.

### Post-hoc analysis of time between diagnoses and socio-economic factors

When analysing the results from the post-hoc test, Table 1 shows the differences between women and men. Women were overall more likely to receive their psychiatric comorbidity diagnosis before the GD diagnosis, while men were more likely to receive their diagnoses concurrently. Drug prescriptions were, when analysed individually, more likely to be prescribed before a diagnosis of gambling disorder in women, and concurrently in men. In total, statistically significant differences were also seen for men receiving a prescription after a GD diagnosis. For socio-economic factors, there was only a statistically significant difference in women receiving sickness/activity/rehabilitation compensation prior to GD diagnosis than men, and for men to receive a compensation after GD diagnosis.

### Analysis of socio-economic factors

**Fig 5:**
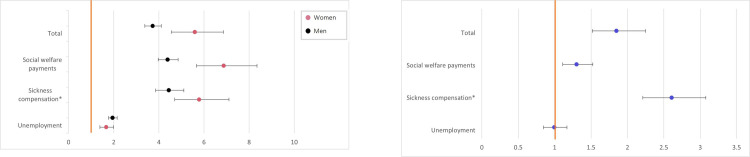
**A.** Odds ratios with 95% CI for social welfare payments, sickness, activity and rehabilitation compensation, and unemployment in the GD group, compared to controls. Orange line accentuates odds ratio for controls (baseline). * Sickness compensation also encompasses activity and rehabilitation compensation. **B.** Odds ratios with 95% CI for social welfare payments, sickness, activity and rehabilitation compensation, and unemployment in women, compared to men in the GD group. Orange line accentuates odds ratio for men (baseline). * Sickness compensation also encompasses activity and rehabilitation compensation.

It was more common for individuals in the GD group to have social welfare payments, sickness/activity/rehabilitation compensation, or to be unemployed, compared to controls (Fig 5A). Overall, women in the GD group were also more likely to be socio-economically vulnerable, compared to men (Fig 5B).

## Discussion

This study found that women with GD overall had higher odds for psychiatric comorbidity, especially affective and anxiety disorders, compared to men. Women were also more likely to have social welfare payments and sickness, rehabilitation or activity compensation. Furthermore, women were overall more likely to receive their psychiatric diagnosis prior to their GD diagnosis, while men were more likely to receive the diagnoses concurrently. For sickness/activity/rehabilitation compensation, women were more likely to receive it after GD diagnosis, whereas men were more likely to receive it before. For other socio-economic factors, no gender differences were seen.

The results in this study are congruent with previous research on the differences of psychiatric disorders in men and women with GD [11], as women in this study have overall higher risk of having a psychiatric disorder alongside the gambling disorder. However, only the subgroups of affective disorders and anxiety disorders, PTSD and phobias (F40-F49) showed a significant difference between genders in the GD group. When analysing psychotic disorders, there was a large spread in odds ratio for women, which might be explained by the small number of individuals with that diagnosis. For alcohol use disorder, the trend points towards men having a higher risk, which has been seen in other studies as well [12]. However, for drug prescriptions, women seem overrepresented in having a treatment for alcohol use disorder, although the results were not statistically significant. The spread in confidence interval was also large between the GD group and controls, probably due to the fact that only 17 women had a prescription targeted at alcohol use disorder in the control group.

When comparing the odds ratios for psychiatric comorbidity with the odds ratios for the drug treatment group, there was also a significant difference between genders in the use of antidepressants, sedatives and hypnotics. The difference was rather small in the antidepressant group, and men in the GD group had a higher odds than women compared to controls, and one explanation to this might be that women without GD also had a rather high prescription rate of antidepressants [13].

For affective disorders, the results were statistically significant for women receiving the diagnosis before GD, and men receiving the diagnoses concurrently. However, for anxiety disorders, there was no significant difference between genders. Earlier studies have shown that anxiety and mood disorders predispose for gambling disorder [7,14]. One reason behind the non-significant results for anxiety disorders in this study might be that the group did not only contain anxiety disorders specifically, but also included PTSD, obsessive-compulsive disorder, phobias, and dissociative syndrome, which could have affected the results. There were also fewer individuals in this group compared to affective disorders, which might be of importance. When analysing patients who received the diagnosis mental disorder, not otherwise specified (F99), again women were more likely to receive the diagnosis prior to GD. The individuals with an F99 diagnosis are a major group in outpatient care, and the diagnosis has been shown to mostly reflect individuals with an affective or anxiety disorder [15]. The true composition of the group in this study is not known, but the significant results for women are in line with those for affective disorders.

For alcohol use disorder, the trend pointed towards women receiving their GD diagnosis first. One possible explanation to this is that in men, alcohol use seems to lead to heavier and potentially more harmful gambling, while in women, alcohol use is more a sign of other mental health issues [16], and in this case, it is possible that women use alcohol as a way to cope with the gambling problems. Interestingly, this pattern was not seen in other substance use disorders, and a larger proportion of both genders seem to receive a substance use disorder diagnosis prior to GD diagnosis. One explanation could be that GD might develop as a consequence of drug use, as substance use disorders increase the risk of GD [17]. One might also speculate that some individuals with substance use disorder might try to finance the costs of their substance use through gambling, although this is a poorly investigated subject.

When comparing time between diagnoses for prescriptions antidepressants, sedatives and hypnotics, this study found that women have a strong tendency towards receiving these prescriptions prior to their GD diagnosis, compared to men. However, these kinds of medications extend to a broader population than the affective and anxiety disorder groups, as for example antidepressants might also be used for other psychiatric conditions [18]. Therefore, the findings should be interpreted with caution.

For socio-economic factors, the odds ratios were higher for women in the GD group to have social welfare payments and sickness/activity/rehabilitation compensation, while there was no difference between genders in unemployment rates. In the general population, it is also more common for women to have social welfare payments and sickness compensation [19,20], but the gender differences for these variables in GD have to our best knowledge not been investigated. An interesting finding from the results is that the difference in unemployment rate in the GD group compared to controls was not as high as for sickness compensation and social welfare payments. One reason behind this might be that unemployment is common even among individuals without GD, ranging between 4.3 and 6.4% between years 2005–2019 in age groups 25–54 years [21]. This study was only able to measure whether someone had been unemployed during any period, therefore it is possible for an individual to have been unemployed only for a short period of time. It would be interesting to further investigate unemployment for prolonged periods of time and see whether there are larger differences in the GD group compared to controls, as well as between genders.

It was more common for women in the GD group to have social welfare payments than men, however for controls, it was more common for men. This data differs from the other socio-economic factors and interestingly, both groups showed statistically significant differences. It is known that the most common type of household to have social welfare payments are men who live alone without children [22], but the gender differences in individuals with GD are poorly investigated. However, it has been shown that receiving social welfare payments is a risk factor for GD [23]. The overall results in this study point towards women with GD being more socio-economically vulnerable than men. When analysing time between socio-economic factors and GD, it was most common for both men and women to have the factors before GD, which could further support the claim that lower socio-economic status predisposes to GD [9]. Gender differences were only seen in sickness/activity/rehabilitation compensation, where men seem to have long time disease more often before GD diagnosis than women, whereas women already had it when they receive their GD diagnosis, much like the results for the analysis of psychiatric comorbidity.

One limitation with this study is that the data in the registers is based on ICD codes, which in turn are diagnoses delivered by health care providers. There is a potential risk in individuals having a psychiatric disorder not being recognised by the health care provider, which has been seen for affective and anxiety disorders in primary care [24]. When analysing whether a patient received their GD diagnosis prior to or after another psychiatric disorder, another limitation is that a person may have a diagnosis long before they receive the diagnosis from their health care provider, possibly because they do not seek help early on [25]. Gender aspects in attitudes towards mental health care should also be taken into consideration, as this could alter the time between diagnoses aspect. Men are not as likely to seek help for their mental health issues, as has been shown in other studies [26], and pathological gambling is not an exception [27], which could potentially mean that when they do seek help, they receive more than one diagnosis in a relatively short time span. This might be the case for the analysis of neuropsychiatric functional limitations, as many of these conditions are likely to emerge earlier in life, however the results in this study showed that men were more likely to receive a neuropsychiatric diagnosis concurrently with GD. Another factor that might alter the time between diagnoses is that GD sometimes might be mistaken or overlooked for another psychiatric diagnosis, such as depression, which was seen in a study performed on women [28], and therefore it is possible that some women actually already are pathological gamblers at the time they receive another psychiatric diagnosis. The factors described above emphasise that the validity of the time aspect between GD and other psychiatric disorders should be taken with great caution, as many factors may play a role in the gender differences.

Another limitation of the study is the lack of confounding adjustment. A multivariate analysis to control for possible confounders, such as age, would have been optimal. In this study, the individuals were pair matched, meaning each case with GD was matched with two controls based on age and sex. However, in the analyses, the individuals with GD were not matched to their respective controls in a matched analysis. Although age and sex distributions were equal in the two groups, this does not guarantee that confounding through the study design is eliminated, and should ideally be controlled in a matched analysis, which was not performed in this study.

Some of the patients included in the study had not had their GD diagnosis more than a couple of months, or a few years, at most. This means that some of the socio-economic and psychiatric factors that have been evaluated in this study may not yet have developed in these individuals, and an even longer perspective of time would be interesting to follow, and maybe exclude individuals who have not had the time to develop any of the investigated variables.

Since the registers used in this study have collected data from a large period of time, namely between 2005 and 2019, gambling habits may have changed, especially with increasing use of the Internet. Studies show that online gambling is more likely to cause GD, than offline gambling [29]. One effect of this might be that patients who develop a GD are increasingly younger individuals. Furthermore, the proportion of problem gamblers has decreased, while the prevalence of the most severe gambling problems (likely corresponding to a GD) has increased from 0.3 to 0.6 percent [30]. However, if this is connected to Internet use is unclear.

One strength of this study is the overall large number of patients included. The registers of individuals with GD used in this study are unique in that they cover all patients with a GD diagnosis in Sweden. It is also a strength that the individuals have been assigned two controls to compare with based on age and gender, who are included at the same time as the individual with GD.

## Conclusions

Psychiatric comorbidity is overall more common in women with GD, than in men. Women were more likely to receive another psychiatric diagnosis before the GD diagnosis, whereas men were more likely to receive the diagnoses concurrently. Women were overall also more socio-economically vulnerable than men. However, further research is required to validate these findings.
